# Folic Acid-Metabolizing Enzymes Regulate the Antitumor Effect of 5-Fluoro-2′-Deoxyuridine in Colorectal Cancer Cell Lines

**DOI:** 10.1371/journal.pone.0163961

**Published:** 2016-09-29

**Authors:** Hiroshi Tsukihara, Kenta Tsunekuni, Teiji Takechi

**Affiliations:** Translational Research Laboratory, Taiho Pharmaceutical Co., Ltd., Tokushima, Japan; Brandeis University, UNITED STATES

## Abstract

In colorectal cancer chemotherapy, the current standard of care includes combination therapy with 5-fluorouracil (5-FU) and leucovorin (LV). However, the factors that determine the LV-mediated enhancement of 5-FU antitumor activity are not fully understood. Therefore, we investigated the roles of thymidine synthase (TYMS), folate receptor 1 (FOLR1), dihydrofolate reductase (DHFR), phosphoribosylglycinamide formyltransferase (GART), methylenetetrahydrofolate dehydrogenase (MTHFD1), and methylenetetrahydrofolate reductase (MTHFR) in LV-mediated enhancement of 5-fluoro-2′-deoxyuridine (FdUrd) cytotoxicity *in vitro* as a model of 5-FU antitumor activity. These genes were downregulated in DLD-1 and HCT116 human colorectal cancer cells by using small-interfering RNA. Reduced expression of *TYMS* mRNA significantly increased FdUrd cytotoxicity by 100- and 8.3-fold in DLD-1 and HCT116 cells, respectively. In contrast, reducing the expression of *FOLR1*, *DHFR*, *GART*, *MTHFD1*, and *MTHFR* decreased FdUrd cytotoxicity by 2.13- to 12.91-fold in DLD-1 cells and by 3.52- to 10.36-fold in HCT116 cells. These results demonstrate that folate metabolism is important for the efficacy of FdUrd. Overall, the results indicate that it is important to clarify the relationship between folate metabolism-related molecules and 5-FU treatment in order to improve predictions of the effectiveness of 5-FU and LV combination therapy.

## Introduction

**Fig 1:**
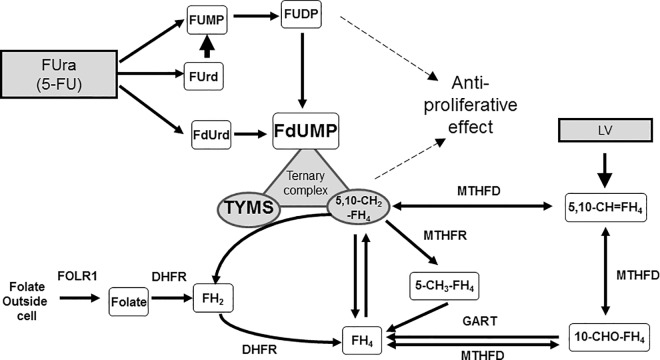
Metabolic pathways of folic acid and mechanism of action of 5-FU. 5-Fluorouracil (5FU) exerts a cytotoxic effect by mediating the formation of an inhibitory ternary complex involving its metabolite 5-fluoro-2-deoxyuridine-5-monophosphate (5FdUMP), thymidylate synthase (TYMS), and 5,10-methylenetetrahydrofolate (5,10-CH_2_-FH_4_). Increased intracellular concentrations of 5,10-CH_2_-FH_4_ enhance the formation and stability of this inhibitory ternary complex, thereby augmenting the cytotoxic effect of 5FU. The amount of 5,10-CH_2_-FH_4_ is regulated by folate receptor 1 (FOLR1), dihydrofolate reductase (DHFR), phosphoribosylglycinamide formyltransferase (GART), methylenetetrahydrofolate dehydrogenase 1 (MTHFD1), and methylenetetrahydrofolate reductase (MTHFR).

Worldwide, colorectal cancer (CRC) was the third most common cancer (9.7%) and the fourth leading cause of cancer-related deaths in 2012 [1]. In CRC chemotherapy, the current standard of care includes combination treatment with 5-fluorouracil (5-FU) and leucovorin (LV). One of the anticancer mechanisms of 5-FU involves the inhibition of thymidylate synthase (TYMS) via formation of a ternary complex between TYMS, 5,10-methylenetetrahydrofolate (5,10‑CH_2_FH_4_), and 5-fluoro-2’-deoxyuridine 5’-monophosphate (FdUMP), which is the active form of 5-FU (Fig 1). Although LV itself has no antitumor activity, it has been used to increase the intracellular concentration of 5,10‑CH_2_FH_4_, thereby increasing the efficacy of 5-FU.

LV enters cells via the reduced folate carrier, where it is metabolized to 5,10‑CH_2_FH_4_ and then polyglutamated by folylpolyglutamate synthetase (FPGS) [2]. Polyglutamation not only increases the cellular retention of 5,10‑CH_2_FH_4_ but also enhances the stabilization of its ternary complex with TYMS and FdUMP [2]. The terminal glutamates of the polyglutamated folates synthesized by FPGS are removed by gamma-glutamyl hydrolase (GGH) [3]. Sakamoto *et al*. [4] found that in DLD-1 cells, suppression of FPGS by siRNA reduced the basal level of reduced folate and the folate level after LV treatment, and enhanced the ability of LV to increase FdUrd-induced cytotoxicity. Similarly, downregulation of *GGH* by siRNA increased the sensitivity of cells to 5-fluoro-2′-deoxyuridine (FdUrd) when combined with LV [4]. These results suggest that expression of both FPGS and GGH within tumor cells controls the ability of LV to enhance the antitumor activity of 5-FU by regulating folate levels.

Verifying the relationship between folic acid metabolism-related molecules and the efficacy of FdUrd *in vitro* is important to understand the contribution of folic acid metabolism to *in vivo* 5-FU-based chemotherapy. To investigate this relationship, we used siRNA to suppress the expression of the following folic acid metabolism-related genes: *TYMS*, folate receptor 1 (*FOLR1*), dihydrofolate reductase (*DHFR*), phosphoribosylglycinamide formyltransferase (*GART*), methylenetetrahydrofolate dehydrogenase 1 (*MTHFD1*), and methylenetetrahydrofolate reductase (*MTHFR*). The effect of downregulating these genes on cell proliferation and the inhibitory effect of FdUrd were then evaluated using two different CRC cell lines *in vitro*.

## Materials and Methods

### Drugs

FdUrd was purchased from Tokyo Chemical Industry (Tokyo, Japan). Leucovorin was purchased from Wako Pure Chemical Industries (Osaka, Japan).

### Cell lines

Two human CRC cell lines (DLD-1 and HCT116) were obtained from the American Type Culture Collection (Manassas, VA, USA). DLD-1 was grown in Roswell Park Memorial Institute 1640 medium (Sigma–Aldrich, St. Louis, MO, USA) with 10% fetal bovine serum (Sigma-Aldrich Japan, Tokyo, Japan), and HCT116 was grown in Dulbecco’s modified Eagle’s medium (Wako Pure Chemical Industries, Osaka, Japan) with 10% fetal bovine serum.

### Cytotoxicity test

Cells were seeded at a density of 2 × 10^3^ (DLD-1) and 1 × 10^3^ (HCT116) cells per well in 96-well plates, incubated overnight, and then treated with the drug for 72 h. Cell numbers were determined using a simplified crystal violet staining method [5]. IC_50_ values, representing the respective concentrations at which 50% of cell growth was inhibited, were calculated from the regression line of concentration-response cytotoxicity plots as the concentration that showed 50% absorbance of the control using XLfit software (ID Business Solutions, Guildford, UK).

### Quantitative reverse-transcription polymerase chain reaction (qRT-PCR)

qRT-PCR was performed on a PRISM^®^ 7900HT sequence detection system (Applied Biosystems, Foster City, CA, USA) using TaqMan^®^ Universal PCR Master Mix (Applied Biosystems). Gene expression levels were normalized to those of β-actin (ACTB). The primers and TaqMan^®^ probes were prepared using Assay-on-Demand gene-expression products (Applied Biosystems) and Human ACTB Endogenous Control (VIC / MGB Probe, Applied Biosystems). Probe IDs were Hs00195560_m1 for *MTHFR*, Hs01068263_m1 for *MTHFD1*, Hs00894582_m1 for *GART*, Hs01124179_g1 for *FOLR1*, Hs00758822_s1 for *DHFR*, and Hs00426586_m1 for *TYMS*.

### Silencing of folic acid metabolism-related genes

The siRNA oligonucleotides for *MTHFR* (ID: HSS106762) and *MTHFD1* (ID: HSS106759) were prepared using Stealth RNAi™ (Thermo Fisher Scientific, Waltham, MA, USA). siRNAs for *GART* (ID: s735) and *FOLR1* (ID: s5330) were prepared using Silencer^®^ siRNA (Thermo Fisher Scientific). siRNA for *DHFR* (ID: SI00299992) was prepared using FlexiTube siRNA (Qiagen, Venlo, The Netherlands). siRNA for *TYMS* was prepared using MISSION^®^ siRNA (Sigma-Aldrich). The sense and antisense strand sequences of *TYMS* siRNA were 5′-CAAUCCGCAUCCAACUAUUTT -3′ and 5′-AAUAGUUGGAUGCGGAUUGTT -3′, respectively. The control siRNA oligonucleotide was Stealth RNAi™ Negative Control Med GC (Thermo Fisher Scientific). Cells were plated into 6-well plates at a density of 2.5 × 10^4^ cells per well, incubated overnight, and then treated with RNAi duplex-Lipofectamine^®^ RNAiMAX (Thermo Fisher Scientific) complexes (final concentration, 10 nM) for 24 h.

### Statistical analyses

Statistical analyses were performed using Student’s *t*-test or Dunnett’s multiple test with JMP (SAS Institute Inc., Cary, NC, USA). P < 0.05 was considered statistically significant.

## Results

### Effect of downregulating *TYMS* on FdUrd cytotoxicity

**Fig 2:**
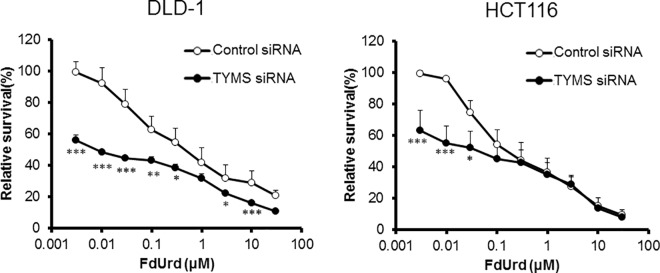
Effect of TYMS silencing on the cytotoxic effect of FdUrd in DLD-1 and HCT116 cells. At 48 h after treatment with control or TYMS siRNA, cells were treated with FdUrd for 72 h. The siRNA significantly enhanced FdUrd toxicity at low concentrations in both cell lines. All data are expressed as the mean ± standard deviation (SD). *p < 0.05, **p < 0.01, ***p < 0.005, statistically significant when compared to a control using Student’s *t*-test.

To examine the influence of *TYMS* expression on the cytotoxicity of FdUrd, we abolished the expression of TYMS in DLD-1 and HCT116 cells using siRNA (S1 Table). Cells treated with control or *TYMS* siRNA were then exposed to increasing concentrations of FdUrd (0.003–30 μM) for 72 h, and survival relative to untreated cells was determined (Fig 2). The sensitivity of cells to FdUrd was increased in *TYMS*-downregulated cells, especially at low concentrations (0.003–0.3 μM). In control siRNA-treated DLD-1 and HCT116 cells, the IC_50_ values of FdUrd were 0.67 and 0.25 μM, respectively. In DLD-1 and HCT116 cells treated with *TYMS* siRNA, the IC_50_ values of FdUrd were 0.01 and 0.03 μM, respectively. The ratios of IC_50_ values in cells treated with control and TYMS siRNA were 67 and 8.3 in DLD-1 and HCT116 cells, respectively (Table 1).

**Table 1:** Change of sensitivity to FdUrd upon silencing of folic acid metabolism-related genes.

	Fold change of IC_50_ versus control	
	DLD-1 cells	HCT116 cells
*TYMS*	0.01	0.12
*FOLR1*	12.9	10.4
*DHFR*	6.18	6.80
*GART*	8.03	9.84
*MTHFD1*	4.07	7.96
*MTHFR*	2.13	3.52

Comparison of IC_50_ values in target siRNA-treated cells and control siRNA-treated cells.

### Effect of downregulating *TYMS* on the enhancement of FdUrd cytotoxicity by LV

**Fig 3:**
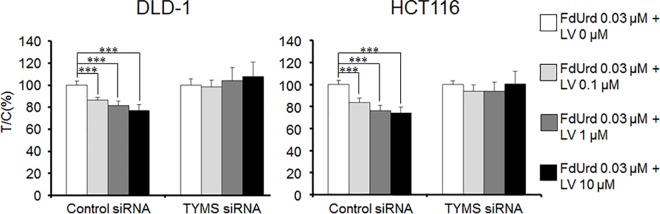
Effect of TYMS silencing on the increased FdUrd cytotoxicity induced by leucovorin (LV). DLD-1 and HCT116 cells were treated with control or TYMS siRNA, and then with 0.03 μM FdUrd plus increasing concentrations of LV for 72 h. LV-enhanced cytotoxicity was prevented by silencing of TYMS. All data are expressed as the mean ± SD (n = 3). ***p < 0.001, statistically significant when compared to a control using Dunnett’s multiple *t*-test.

We next examined the influence of TYMS on LV-enhanced FdUrd cytotoxicity. LV significantly enhanced FdUrd cytotoxicity in a concentration-dependent manner (0.1–10 μM) and at all concentrations in both DLD-1 and HCT116 cells treated with control siRNA (*p* < 0.01). The relative survival of cells treated with 0.03 μM FdUrd and 0.1, 1, or 10 μM LV was 86.6, 81.5, and 77.0% in DLD-1 cells, and 83.7, 75.1, and 72.1% in HCT116 cells, respectively. In contrast, LV did not enhance FdUrd cytotoxicity in *TYMS*-downregulated DLD-1 or HCT116 cells at any concentration (Fig 3).

### Effect of downregulating *MTHFR*, *MTHFD1*, *DHFR*, *GGH*, and *GART* on FdUrd cytotoxicity

**Fig 4:**
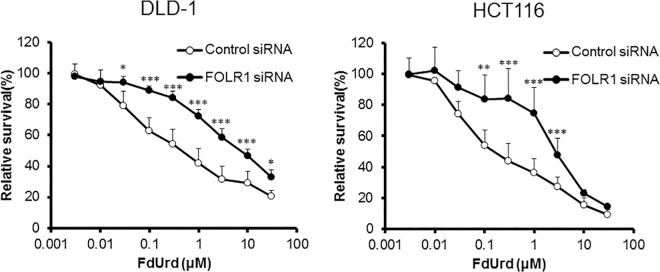
Effect of folate receptor 1 (*FOLR1*) silencing on FdUrd cytotoxicity. At 48 h after treatment of DLD-1 and HCT116 cells with control or FOLR1 siRNA, cells were treated with FdUrd for 72 h and cell viability was determined. Silencing of *FOLR1* reduced the efficacy of FdUrd at 0.03–30 μM in DLD-1 cells and at 0.03–3 μM in HCT116 cells. All data are expressed as the mean ± SD. *p < 0.05, **p < 0.01, ***p < 0.005, statistically significant when compared to a control using Student’s *t*-test.

**Fig 5:**
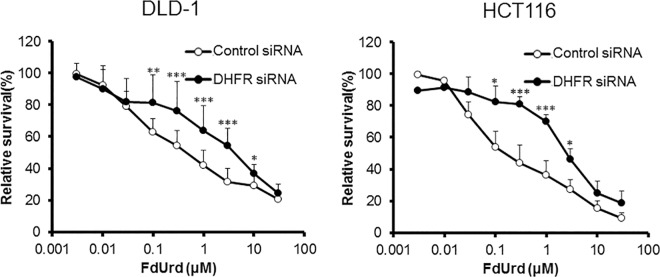
Effect of dihydrofolate reductase (*DHFR*) silencing on FdUrd cytotoxicity. At 48 h after treatment of DLD-1 and HCT116 cells with control or DHFR siRNA, cells were treated with FdUrd for 72 h, and cell viability was determined. Silencing of *DHFR* reduced the efficacy of FdUrd at 0.1–10 μM in DLD-1 cells and at 0.1–3 μM in HCT116 cells. All data are expressed as the mean ± SD. *p < 0.05, **p < 0.01, ***p < 0.005, statistically significant when compared to a control using Student’s *t*-test.

**Fig 6:**
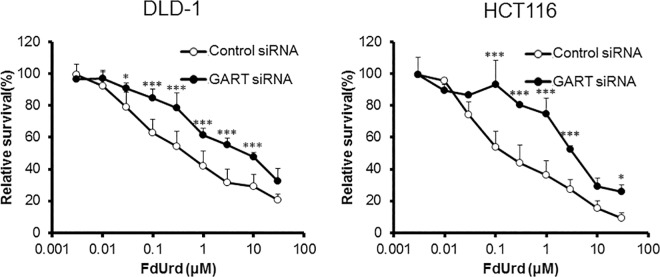
Effect of phosphoribosylglycinamide formyltransferase (*GART*) silencing on FdUrd cytotoxicity. At 48 h after treatment of DLD-1 and HCT116 cells with control or GART siRNA, cells were treated with FdUrd for 72 h, and cell viability was determined. Silencing of *GART* reduced the efficacy of FdUrd at 0.03–10 μM in DLD-1 cells and at 0.1–3 and 30 μM in HCT116 cells. All data are expressed as the mean ± SD. *p < 0.05, ***p < 0.005, statistically significant when compared to a control using Student’s *t*-test.

**Fig 7:**
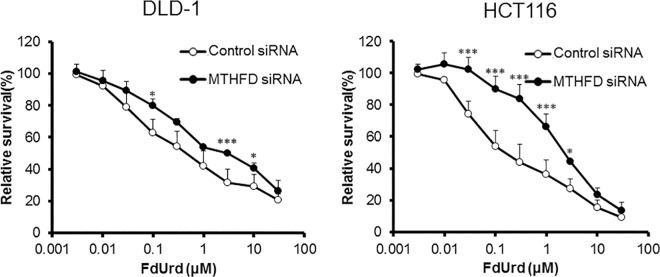
Effect of methylenetetrahydrofolate dehydrogenase 1 (*MTHFD1*) silencing on FdUrd cytotoxicity. At 48 h after treatment of DLD-1 and HCT116 cells with control or MTHFD1 siRNA, cells were treated with FdUrd for 72 h, and cell viability was determined. Silencing of *MTHFD1* reduced the efficacy of FdUrd at 3–10 μM in DLD-1 cells and at 0.03–3 μM in HCT116 cells. All data are expressed as the mean ± SD. *p < 0.05, ***p < 0.005, statistically significant when compared to a control using Student’s *t*-test.

**Fig 8:**
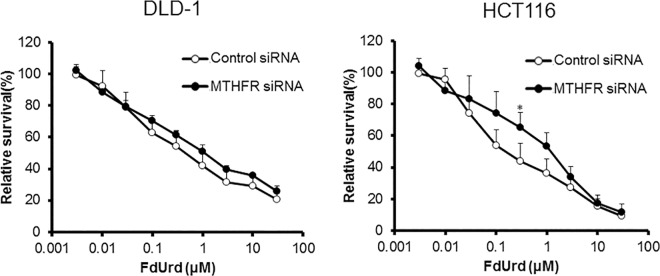
Effect of *MTHFR* silencing on FdUrd cytotoxicity. At 48 h after treatment of DLD-1 and HCT116 cells with control or MTHFR siRNA, cells were treated with FdUrd for 72 h, and cell viability was determined. Silencing of *MTHFR* reduced the efficacy of FdUrd at 0.3 μM in HCT116 cells. All data are expressed as the mean ± SD. *p < 0.05, statistically significant when compared to a control using Student’s *t*-test.

To investigate the influence of the supply of 5,10-CH_2_-FH_4_ on FdUrd cytotoxicity, we downregulated folic acid-metabolizing enzymes and evaluated the sensitivity of DLD-1 and HCT116 cells to FdUrd. In DLD-1 cells, downregulation of folic acid-metabolizing enzymes, except for MTHFR, reduced the efficacy of FdUrd. The IC_50_ values of cells with downregulated *FOLR1*, *DHFR*, *GART*, and *MTHFD1* were 8.65, 4.14, 5.38, and 2.73 μM, respectively. The IC_50_ value of DLD-1 cells treated with control siRNA was 0.67 μM. In HCT116 cells, siRNA against *FOLR1*, *DHFR*, *GART*, *MTHFD1*, and *MTHFR* also reduced the sensitivity to FdUrd with IC_50_ values of 2.59, 1.70, 2.46, 1.99, and 0.88 μM, respectively, compared to the control siRNA IC_50_ value of 0.25 μM (Figs 4–8). The ratios of the changes of IC_50_ values in cells treated with each siRNA compared to the control siRNA treated cells were listed in Table 1.

## Discussion

To investigate the influence of folic acid-metabolizing enzymes on the efficacy of FdUrd, the active form of 5-FU, we downregulated the corresponding genes by using siRNA. Suppression of *TYMS* enhanced the efficacy of FdUrd in human colorectal tumor cells. In contrast, suppression of *GART*, *DHFR*, *MTHFD1*, and *FOLR1* in DLD-1 cells and *GART*, *DHFR*, *MTHFD1*, *FOLR1*, and *MTHFR* in HCT116 cells decreased the efficacy of FdUrd.

Balancing of the amounts of FdUMP, TYMS, and reduced folate in cells appears to be important for the inhibition of TYMS through the formation of a ternary complex [6]. Interestingly, the enhanced anti-proliferative effect of FdUrd after downregulation of *TYMS* was more remarkable at low concentrations (< 0.3 μM) than at high concentrations (>0.3 μM) in both HCT116 and DLD-1 cells. At low FdUrd concentrations, because the amount of folate in the medium was limited, TYMS may have been inhibited effectively by the formation of a ternary complex when *TYMS* expression was downregulated. However, when *TYMS* expression was high, the amount of folate in the medium may have been insufficient to create ternary complexes and inhibit TYMS effectively. We investigated the relationship between the concentration of LV and the efficacy of FdUrd. In cells treated with control siRNA, addition of LV enhanced the cytotoxicity of FdUrd, whereas downregulation of *TYMS* abolished this effect (Fig 3). As expected, when TYMS expression was high (i.e., with control siRNA), additional LV was needed to effectively inhibit TYMS, whereas following treatment with TYMS siRNA, additional LV was not necessary. Tsujimoto *et al*. reported that the growth inhibitory effect of tegafur-uracil plus LV was superior to that of tegafur-uracil alone in tumors possessing high TYMS activity [7]. Similarly, our data show that the level of TYMS expression is important to determine whether additional LV is necessary to enhance the cytotoxicity of FdUrd. O’Dwyer et al. reported that addition of LV increased the side effects of 5-FU in conjunction with enhanced efficacy [8]. Thus, minimizing the dose of LV required to inhibit TYMS by forming a ternary complex is considered a desirable aspect of combined 5-FU and LV treatment. Because the available folates may be saturated in tumors with low TYMS expression, thereby limiting the formation of ternary complexes even at low doses of LV, controlling the amount of LV administered in relation to the tumor folate level may improve the balance between the safety and efficacy of 5-FU treatment.

FOLR1 imports folic acid into cells and increases the folate pool (Fig 1). Karasawa *et al*. reported that expression of *FOLR1* was decreased in a 5-FU-resistant human colon cancer cell line compared to its parental cell line, DLD-1 [9]. Consistent with their results, we found that the anti-proliferative efficacy of FdUrd was decreased by downregulating *FOLR1* (Fig 4). These results suggest that decreasing the amount of FOLR1 reduces the folate pool and inhibits the formation of ternary complexes.

Kalmbach *et al*. reported a del/del polymorphism in *DHFR* that diminished the capacity of the enzyme to reduce folic acid and limited the assimilation of folic acid into cellular folate stores at both high and low folic acid intakes [10]. Consistent with their results, our data showed that downregulation of *DHFR* suppressed the efficacy of FdUrd (Fig 5). Our findings suggest that abnormal folate metabolism resulting from inefficient functioning of DHFR, which synthesizes FH_4_ by reducing FH_2_ (Fig 1), affects the folate pool and results in limited folic acid assimilation.

Some studies have reported associations between *MTHFR* polymorphisms that reduced enzyme activity and the clinical outcome in CRC patients [11–13], whereas others have not [14–16]. In our study, suppression of *MTHFR* expression reduced the efficacy of FdUrd in HCT116 cells (Fig 8). Because MTHFR converts 5,10-CH_2_-FH_4_ to 5-CH_3_-FH_4_, downregulation of this enzyme may increase the formation of the ternary complex (Fig 1). However, Kawakami *et al*. reported that 5,10-CH_2_-FH_4_ and FH4 levels were lower in tumors with *MTHFR* polymorphisms [17]. Similar to the *DHFR* del/del polymorphism, the inefficient functioning of MTHFR affects the folate pool, decreasing its ability to assimilate folic acid.

GART and MTHFD1 are involved in folic acid metabolism. However, to our knowledge, no previous study on GART and MTHFD1 has linked them to the 5-FU response. We found a relationship between the efficacy of FdUrd and the expression of both GART and MTHFD1. Similar to *FOLR1*, *DHFR*, and *MTHFR*, downregulation of *GART* or *MTHFD1* suppressed the efficacy of FdUrd (Figs 6 and 7).

Except for *TYMS*, downregulation of the folic acid-metabolizing enzymes that we investigated decreased the efficacy of FdUrd. Because downregulation of folic acid metabolizing enzymes results in reduction and imbalance of the folate pool, these results were expected. However, our study was limited to *in vitro* experiments using CRC cell lines. To confirm the relationship between the efficacy of 5-FU and folate metabolism-related molecules, a clinical study is necessary. Importantly, based on our data, further investigation of all factors evaluated in this study is warranted.

In conclusion, the results of the current study indicated that clarifying the relationship between folate metabolism-related enzymes and the efficacy of 5-FU treatment is important for predicting the effect of a combination therapy involving 5-FU and LV. With an improved understanding of this relationship, better therapy for CRC using 5-FU can be designed.

## Supporting Information

S1 Table Efficiency of silencing of folic acid metabolism-related genes by siRNA. The expression of mRNA was evaluated at different time points after transfection with siRNA. Data are presented as percentages of fold changes of mRNA expression following application of gene-specific siRNA relative to control siRNA.
(DOCX)

## References

[1] Ferlay J, Soerjomataram I, Dikshit R, Eser S, Mathers C, Rebelo M, et al Cancer incidence and mortality worldwide: Sources, methods and major patterns in GLOBOCAN 2012. Int J Cancer. 2015;136: E359–E386. 10.1002/ijc.29210 25220842

[2] Longley DB, Harkin DP, Johnston PG. 5-fluorouracil: mechanisms of action and clinical strategies. Nat Rev Cancer. 2003;3: 330–338. 10.1038/nrc1074 12724731

[3] Carmichael J, Popiela T, Radstone D, Falk S, Borner M, Oza A, et al Randomized comparative study of tegafur/uracil and oral leucovorin versus parenteral fluorouracil and leucovorin in patients with previously untreated metastatic colorectal cancer. J Clin Oncol. 2002;20: 3617–3627. 10.1200/jco.2002.10.129 12202662

[4] Sakamoto E, Tsukioka S, Oie S, Kobunai T, Tsujimoto H, Sakamoto K, et al Folylpolyglutamate synthase and γ-glutamyl hydrolase regulate leucovorin-enhanced 5-fluorouracil anticancer activity. Biochem Biophys Res Commun. 2008;365: 801–807. 18035049 10.1016/j.bbrc.2007.11.043

[5] Saotome K, Morita H, Umeda M. Cytotoxicity test with simplified crystal violet staining method using microtitre plates and its application to injection drugs. Toxicol In Vitro. 1989;3: 317–21. 10.1016/0887-2333(89)90039-8 20702298

[6] Spears CP, Gustavsson BG, Berne M, Frösing R, Bernstein L, Hayes AA. Mechanism of innate resistance to thymidylate synthase inhibition after 5-fluorouracil. Cancer Res. 1988;48: 5894–5900. 3167844

[7] Tsujimoto H, Tsukioka S, Ono S, Sakamoto E, Sakamoto K, Tsuta K, et al Effect of leucovorin on the antitumor efficacy of the 5-FU prodrug, tegafur-uracil, in human colorectal cancer xenografts with various expression levels of thymidylate synthase. Oncol Lett. 2010;1: 973–980. 10.3892/ol.2010.172 22870097 PMC3412464

[8] O'Dwyer PJ, Manola J, Valone FH, Ryan LM, Hines JD, Wadler S, et al Fluorouracil modulation in colorectal cancer: lack of improvement with N -phosphonoacetyl- l -aspartic acid or oral leucovorin or interferon, but enhanced therapeutic index with weekly 24-hour infusion schedule—an Eastern Cooperative Oncology Group/Cancer and Leukemia Group B Study. J Clin Oncol. 2001;19: 2413–2421. 11331320 10.1200/JCO.2001.19.9.2413

[9] Karasawa H, Miura K, Fujibuchi W, Ishida K, Kaneko N, Kinouchi M, et al Down-regulation of cIAP2 enhances 5-FU sensitivity through the apoptotic pathway in human colon cancer cells. Cancer Sci. 2009;5: 903–913. 10.1111/j.1349-7006.2009.01112.x PMC11159709 19302291

[10] Kalmbach RD, Choumenkovitch SF, Troen AP, Jacques PF, D'Agostino R, Selhub J. A 19-base pair deletion polymorphism in dihydrofolate reductase is associated with increased unmetabolized folic acid in plasma and decreased red blood cell folate. J Nutr. 2008;138: 2323–2327. 10.3945/jn.108.096404 19022952 PMC2855991

[11] Cohen V, Panet-Raymond V, Sabbaghian N, Morin I, Batist G, Rozen R, et al Methylenetetrahydrofolate reductase polymorphism in advanced colorectal cancer: a novel genomic predictor of clinical response to fluoropyrimidine-based chemotherapy. Clin Cancer Res. 2003;9: 1611–1615. 12738713

[12] Etienne MC, Ilc K, Formento JL, Laurent-Puig P, Formento P, Cheradame S, et al Thymidylate synthase and methylenetetrahydrofolate reductase gene polymorphisms: relationships with 5-fluorouracil sensitivity. Br J Cancer. 2004;90: 526–534. 10.1038/sj.bjc.6601523 14735204 PMC2409555

[13] Marcuello E, Altés A, Menoyo A, Rio ED, Baiget M. Methylenetetrahydrofolate reductase gene polymorphisms: genomic predictors of clinical response to fluoropyrimidine-based chemotherapy? Cancer Chemother Pharmacol. 2006;57: 835–840. 10.1007/s00280-005-0089-1 16187112

[14] Gusella M, Frigo AC, Bolzonella C, Marinelli R, Barile C, Bononi A, et al Predictors of survival and toxicity in patients on adjuvant therapy with 5-fluorouracil for colorectal cancer. Br J Cancer. 2009;100: 1549–1557. 10.1038/sj.bjc.6605052 19384296 PMC2696766

[15] Afzal S, Jensen SA, Vainer B, Vogel U, Matsen JP, Sørensen JB, et al MTHFR polymorphisms and 5-FU-based adjuvant chemotherapy in colorectal cancer. Ann Oncol. 2009;20: 1660–1666. 10.1093/annonc/mdp046 19465420

[16] Glimelius B, Garmo H, Berglund A, Fredriksson LA, Berglund M, Kohnke H, et al Prediction of irinotecan and 5-fluorouracil toxicity and response in patients with advanced colorectal cancer. Pharmacogenomics J. 2011;11: 61–71. 10.1038/tpj.2010.10 20177420 PMC3036798

[17] Kawakami K, Ruszkiewicz A, Bennett G, Moore J, Watanabe G, Iacopetta B. The folate pool in colorectal cancers is associated with DNA hypermethylation and with a polymorphism in methylenetetrahydrofolate reductase. Clin Cancer Res. 2003;9: 5860–5865. 14676107

