# Supplementary material for: Folic Acid-Metabolizing Enzymes Regulate the Antitumor Effect of 5-Fluoro-2′-Deoxyuridine in Colorectal Cancer Cell Lines
Source: PLoS One. 2016 Sep 29;11(9):e0163961. doi: 10.1371/journal.pone.0163961 (PMC5042458; doi:10.1371/journal.pone.0163961)
Supplement: S1 Table — The expression of mRNA was evaluated at different time points after transfection with siRNA. Data are presented as percentages of fold changes of mRNA expression following application of gene-specific siRNA relative to control siRNA. (DOCX) [file pone.0163961.s001.docx]

**Supporting information**

**S1 Table.**

|  | DLD-1 | | | HCT116 | | |
| --- | --- | --- | --- | --- | --- | --- |
|  | 24 h | 48 h | 120 h | 24 h | 48 h | 120 h |
| *TS* | 4.8 | 10.6 | 18.8 | 15.6 | 14.8 | 58 |
| *FOLR1* | 12.1 | 9.1 | 15.3 | 17 | 10.3 | 49.2 |
| *DHFR* | 8 | 8.5 | 32.4 | 13.5 | 17.2 | 92.4 |
| *GART* | 12.7 | 18.9 | 26.4 | 7.9 | 7.1 | 65.5 |
| *MTHFD1* | 7.5 | 9.7 | 15.2 | 9.7 | 15.1 | 49.8 |
| *MTHFR* | 30.6 | 25.4 | 42.5 | 32.3 | 48.7 | 67.8 |
